# Effectiveness and cost-effectiveness of telehealth in rural and remote emergency departments: a systematic review protocol

**DOI:** 10.1186/s13643-020-01349-y

**Published:** 2020-04-17

**Authors:** Christina Tsou, Suzanne Robinson, James Boyd, Andrew Jamieson, Robert Blakeman, Kylie Bosich, Justin Yeung, Stephanie Waters, Delia Hendrie

**Affiliations:** 1 Curtin University, Kent Street, Bentley, Perth, WA 6102 Australia; 2 La Trobe University, Plenty Rd & Kingsbury Dr, Bundoora, VIC 3086 Australia; 3 WA Country Health Service, 189 Wellington Street, Perth, WA 6000 Australia; 4 Consumer and Community Health Research Network, Level 6, 6 Verdun Street, Nedlands, WA 6009 Australia; 5 WA Country Health Service (Command Centre), Royal Perth Hospital, Level 6, Wellington Street, Perth, WA 6000 Australia; 6 WA Country Health Service, Royal Perth Hospital, Level 6, Wellington Street, Perth, WA 6000 Australia

**Keywords:** Telehealth, Telemedicine, Clinical effectiveness, Treatment outcome, Cost-effectiveness, Economic evaluation, Rural population, Rural health, Remote

## Abstract

**Background:**

Emergency telehealth has been used to improve accessibility of rural and remote patients to specialist care. Evidence to date has demonstrated effectiveness and cost-effectiveness of telehealth in rural and remote emergency departments within a variety of contexts. However, systematic reviews to date have not focused on the rural and remote emergency departments. The purpose of this study is to review the outcome measures used in evaluations of emergency telehealth in rural and remote settings and assess evidence relating to their effectiveness and cost-effectiveness.

**Methods:**

Randomised controlled trials, non-randomised controlled trials, and full and partial economic evaluations (e.g. cost-effectiveness, cost-benefit, and cost-utility analyses) of telehealth in rural and remote emergency departments will be included. Comprehensive literature searches will be conducted in multiple electronic databases (from 1990 onwards): MEDLINE (Ovid), Cochrane Library, Scopus, CINAHL, ProQuest, EconLit, CRD databases (e.g. NHS Economic Evaluation database), and Tufts Cost-Effectiveness Registry. Two authors will independently screen all citations, full-text articles, and abstract data. The methodological quality (or risk of bias) of individual studies will be appraised using an appropriate tool. A systematic narrative synthesis will be provided with information presented in the text and tables to summarise and explain the characteristics and findings of the studies. If feasible, we will conduct random effects meta-analysis.

**Discussion:**

This review will identify gaps in the current body of evidence relating to the effectiveness and cost-effectiveness of rural and remote emergency telehealth services. By confining to articles written in the English language, this analysis may be subjected to publication bias and results need to be interpreted accordingly. We believe the results of this review could be valuable for the design of future economic evaluations of emergency telehealth services implemented in the rural and remote context.

**Systematic review registration:**

PROSPERO CRD42019145903

## Background

Small rural and remote hospitals need to have access to specialty care to ensure that patients receive the right care in the right place at the right time. However, small and rural hospitals face particular challenges related to shortages of primary care and specialist providers. Emergency telehealth services have been used to address this issue [1] and have been widely adopted in resource-poor emergency departments such as those in rural and remote locations [1–4]. Cost-effectiveness studies to date have reported evidence in rural and remote medical emergencies within a wide variety of contexts; however, systematic reviews on effectiveness and cost-effectiveness of telehealth in the emergency departments to date have not focused on the rural and remote settings [5].

A literature review on the impact and effectiveness of emergency telehealth services provided an overview of the suitability, effectiveness, and available evidence on economic outcomes up to September 2013. From 38 articles reviewed, the authors provided an overview of major findings relating to technical quality, user perceptions, clinical processes and outcomes, disposition and throughput, and economic outcomes [6]. However, specific outcome measures to assess the effectiveness of emergency telehealth were not systematically reviewed. A small number of studies reported economic analyses, and no formal cost-effectiveness evaluation was performed in any of the studies reviewed. The studies included in this review have not always used the term ‘cost-effectiveness’ strictly not within the meaning of formal economic evaluations [7]. Since this review, the cost-effectiveness of telehealth in the rural and remote emergency settings have been reported in the context of pre-hospital patient care enabled by telehealth [8, 9], telemetry for chest pain patients presenting to the emergency departments [10], and acute ischemic stroke presentations to rural hospitals [11].

Reported benefits of emergency telehealth in rural and remote settings are not confined to acutely critical presentations. A systematic review published in 2019 on non-critical emergency presentations in the rural and remote emergency departments found that there was potential for telehealth programmes to assist in reducing unnecessary patient transfer and secondary over-triage [12]. These programmes may also increase the capacity of emergency department staff to diagnose and manage patients locally, translating to local hospital admission and reduced discharge rates following teleconsultation [12]. It is unknown whether these findings are transferable to critical emergency presentations. Whether the effectiveness and cost-effectiveness on improving patient outcome is dependent on presenting conditions also remains unanswered.

A global review of telehealth for acute and chronic teleconsultations have identified the impossibility of making generalisations about the clinical and economic effectiveness of telehealth consultations [5]. This review has sourced 29 studies on TeleStroke and 21 studies on emergency care telehealth specialist consultations. The review concluded that emergency care decreases time from presentation to decision, reduces ED time, and increases appropriate transfers and admissions [5]. Despite the recency and comprehensiveness of the review, the analysis on clinical effectiveness and cost-effectiveness across specialty or condition was insufficient, and the lack of rural and remote focus means the context around the effectiveness and cost-effectiveness of the studies reviewed was absent. Time is of the essence in emergency departments particularly in rural and remote settings where longer transfer time is expected if inter-hospital movement is required. Treatment delays in transit due to distance can potentially impact on clinical effectiveness in emergency departments particularly in remote locations, and the extent of telehealth effectiveness in bridging this gap across condition groups has also not been reviewed.

The challenges in using available effectiveness and cost-effectiveness evidence to inform resource allocation decisions is the variations in the clinical effectiveness and outcome measures. This is in turn influenced by the variations in the requirements of each clinical specialty (e.g. mental health, emergency medicine, neurology, ophthalmology, plastic surgery) or condition groups (e.g. acute stroke, acute myocardial infarction, or burns). For example, the proportion of patients receiving the time-critical thrombolysis infusion (tPA) and the modified Rankin Scale (mRS) has been used consistently across most TeleStroke studies as the clinical effectiveness measure; however, tPA infusion is only applicable to ischaemic stroke patients. The effectiveness and cost-effectiveness findings are therefore not transferable to the haemorrhagic stroke and the transient ischaemic attack (TIA) presentations. The determination of stroke subtypes is heavily dependent on whether the site of presentation has computed tomography (CT) capability; therefore, the findings from the TeleStroke are also difficult to generalise to the patient cohorts presenting to hospitals with no CT imaging facility. The variations in telehealth utilisation depend on what conditions patients are presented with and the expected impact of timely specialist consultation on clinical effectiveness. Some cost-effectiveness studies have used incremental cost-effectiveness ratio (ICER) comparing the cost of improving one quality of life year (QALY) [13]. A discussion is pending on the utility and meaning of QALY for emergency telehealth in the rural and remote hospitals, especially variations in its meaning and utility across different clinical categories of presentations.

In order to inform the design of future economic evaluations of emergency telehealth in rural and remote setting, a systematic review is warranted to explore the clinical effectiveness measures used in past evaluations of emergency telehealth implemented in rural and remote settings, the utility of QALY as an outcome measure in the cost-effectiveness studies in conjunction with the methodology, and findings from economic analysis of such services.

The purpose of this study is to review the outcome measures used in evaluations of emergency telehealth in rural and remote settings and to assess evidence relating to their effectiveness and cost-effectiveness.

## Methods/design

This study protocol for a systematic review of the effectiveness and cost-effectiveness of telehealth in rural and remote emergency departments is being reported in accordance with the reporting guidance the Preferred Reporting Items for Systematic Reviews and Meta-Analyses Protocols (PRISMA-P) statement [14, 15] (see PRISMA-P checklist in Additional file 1). This protocol was registered within the International Prospective Register of Systematic Reviews (PROSPERO) (registration number CRD42019145903; https://www.crd.york.ac.uk/PROSPERO/display_record.php?RecordID=145903).

### Eligibility criteria

Studies will be selected according to criteria around the Population, Intervention, Comparator, Outcome(s) of interest, and Study design (PICOS framework) [16]. These are detailed as follows:

- Participants: We will include studies involving patients attended in rural or remote emergency departments. Rural and remote in this review will be defined loosely as presentation locations not within a metropolitan city. Selection of study will not be based on distance between tertiary centre and site of presentation.
- Interventions and comparators: Telehealth technology can range from telephone compared to face to face consults (that is treatment as usual) to video conference consult compared to telephone consults. Studies focusing on the effectiveness of a single mobile device, electronic health records will also be excluded.
- Outcome(s) of interest: The primary outcome will be the incremental cost-effectiveness ratio in terms of costs per life years gained, quality-adjusted life years gained, or disability-adjusted life years avoided. The secondary outcomes will be health outcomes, costs, or both as reported by investigators of included studies. Studies describing the effectiveness of telehealth without a well-defined effectiveness measure will not be included.
- Study design: Eligible studies will be randomised controlled trials, non-randomised controlled trials, and full and partial health economic evaluations. Full economic evaluations will include cost-effectiveness analyses, cost-benefit analyses, cost-utility analyses, and cost minimization analyses. Partial economic evaluations will include cost comparisons, cost analysis, cost-outcome description, cost descriptions, and cost of illness studies. We will exclude reviews, editorials/commentaries, and methodological articles. No limitations will be imposed on publication status (articles, study reports, and unpublished studies will be eligible for inclusion).

The selection criteria have been summarised in Table 1, and only studies published in the English language from 1990 to the present will be considered.

**Table 1:** Selection criteria

Parameter	Inclusion criteria	Exclusion criteria
Population	Rural and remote populations being treated in ED	Rural and remote populations being treated outside of the emergency department
Intervention and comparator	Emergency telehealth vs. treatment as usual could be: • Telephone vs. face to face consults • Video conference vs. telephone consults	Descriptive studies without comparators Study focused on a mobile device or electronic health records
Outcomes	Primary outcome: incremental cost-effectiveness, quality-adjusted life years, or disability adjusted life years avoided. Secondary outcomes: health outcomes, costs, or both as reported by investigators of included studies.	Descriptive statistics without a well-defined effectiveness measure.
Study design	Full and partial economic evaluation Qualitative research Randomised controlled trials Non-randomised controlled trials	Commentaries Expert opinions Government reports Strategic documents Single case reports

### Information sources

The primary source of literature will be a structured search of major electronic databases (from January 1990 onwards): MEDLINE (Ovid), Cochrane Library, Scopus, CINAHL, ProQuest, EconLit, CRD databases (e.g. NHS Economic Evaluation database), and Tufts Cost-Effectiveness Registry. We will perform hand-searching of the reference lists of included studies, relevant reviews, national clinical practice guidelines, or other relevant documents. Content experts and authors who are prolific in the field will be contacted.

#### Search strategy

The search strategy was developed together with the Health Sciences librarian at the Curtin University to improve search quality. A search strategy using medical subject headings (MeSH) was designed to target four key domains: telehealth/telemedicine, emergency department/emergency department, effectiveness, or cost-effectiveness analysis. A draft search strategy for MEDLINE is provided in Additional file 2. The final search strategies will be reported in the complete review. Pilot searches were undertaken for each domain and combined concepts to ensure that the search strategy was effective.

### Study records

#### Data management

Identified records will be managed using the EndNote reference manager, which will enable duplication of records to be identified and removed and records tracked through the screening and data collection process.

#### Selection process

Titles and abstracts will be screened by one reviewer (CT). Articles meeting the selection criteria will be retained for independent assessment against selection criteria by the first reviewer and confirmed by two other reviewers (SR and DH). Any discrepancies will be reassessed by a fourth reviewer (JB) with the remaining discrepancies resolved by the full team. The final list of articles will be downloaded in full text for detailed review. Additional records will be searched from reference lists of the articles retained.

A PRISMA flowchart will be used to demonstrate the process of identification and screening of articles to include in the systematic review, with reasons for exclusion noted.

#### Data extraction

Record extraction will be conducted by one reviewer (CT) and checked by a second reviewer (DH). Any disagreement will be resolved through discussion or through an assessment by a third reviewer (SR). Data will be extracted using a standardised form. For all studies, items to be included in the data extraction sheet are the following: bibliographic information, aim and objectives, country, participant characteristics, intervention and comparator details, outcome measure(s) including clinical effectiveness, and service utilisation indicators. For the economic evaluation studies, additional data items will include perspectives of economic analysis, cost items included, decision-analytic models used, sensitivity analysis performed, and results (Additional file 2).

#### Reporting quality in individual studies

The reporting quality in full economic evaluations will be evaluated using the Consolidated Health Economics Evaluation Reporting Standards (CHEERS) statement. The risk of bias of individual studies will be evaluated using appropriate tools. For randomised control trials, the Risk of Bias 2.0 tool will be used [17], for non-randomised control trials, the ROBINS-I tool [18], and for economic evaluations, the Joanna Briggs Institute (JBI) suite of critical appraisal tools [19].

### Data synthesis

#### Review characteristics and result summaries

A narrative description of the PICO element and findings of included studies will be tabulated in four different tables summarising their bibliographic information, intervention, outcome measures, and conclusions at the study level. Additional file 4 lists the column headings of each of the summary tables. Articles will also be categorised and counted in a two-way matrix according to specialties and operational use of the intervention. An initial matrix is shown in Additional file 5 which will also be used for the collection of outcome measures to assess the effectiveness of emergency telehealth services.

A second-tier description of outcome measures will then be collated and categorised into clinical effectiveness or service utilisation measures, and any validity or data quality issues of the measures will be reviewed. The frequencies of each outcome measure will be counted, and any pattern around the context in which each of the measures has been used will be noted including any data collection issues. The frequency of each of the measures used by each study perspectives (health system or societal perspectives) will be counted, rationales discussed by the authors in relation to the outcome measure will be noted, and appropriateness for economic evaluation will be discussed.

Economic evaluations will be further summarised to include specialty, the type of economic evaluation conducted, effectiveness measure, cost items, and data collection methods including whether the costs are collected from routine administrative data collection, survey samples, or from other sources. The incremental cost-effectiveness ratio (ICER) will be identified from cost-effectiveness studies.

#### Quantitative synthesis

The data from each paper summarised above will be used to build evidence tables of an overall description of included studies. If feasible and appropriate, data points from primary observational studies will be used to perform random effects meta-analyses. Since heterogeneity is expected a priori, we will estimate summary estimates (e.g. mean differences, standard mean differences) and its 95% confidence interval using the Hartung-Knapp-Sidik-Jonkman [20] random effects model. The random effects model assumes the study prevalence estimates to follow a normal distribution, considering both within-study and between-study variations. Forest plots will be used to visualise the extent of heterogeneity among studies. We will quantify statistical heterogeneity by estimating the variance between studies using *I*^2^ statistics. *I*^2^ is the proportion of variation in prevalence estimates that is due to genuine variation in prevalence rather than sampling (random) error. *I*^2^ ranges between 0 and 100% (with values of 0–25% and 75–100% taken to indicate low and considerable heterogeneity, respectively). We will also report Tau2 and Cochran *Q* test with *P* value of < 0.005 considered statistically significant (heterogeneity).

#### Additional analysis

A subgroup analysis of telehealth effectiveness is planned for each specialty to be reported, and a separate subgroup analysis will be performed for economic studies where the outcome is incremental cost-effectiveness in cost/QALY gained. To assess the extent of publication bias, a funnel plot will be used with the effect size on the *x*-axis and total sample size on the *y*-axis. An upside-down funnel-shaped distribution of studies indicates the absence of a publication bias. Publication bias can be suspected if the studies showed an asymmetrical distribution [20]. If publication bias is detected, the trim-and-fill method can be used to correct the bias [21].

## Discussion

Although there is some evidence on the effectiveness of emergency telehealth services, evidence on their effectiveness and cost-effectiveness in rural and remote settings is limited. A closer examination of outcomes used in rural and remote emergency telehealth is an important preparatory step in the design of a robust economic evaluation to capture the impact of distance and remoteness context. This review will provide insight into what outcome measures have been used to assess the effectiveness of rural and remote telehealth services and how the selection of measures impact on the quality of and findings from economic evaluations.

By assessing economic evidence extracted from this review, any critical gaps in the current body of evidence in this field will be identified. Additionally, summarising the effectiveness and cost-effectiveness of emergency telehealth services in the rural and remote context will provide useful information to inform both the design of future economic evaluations and decision-making surrounding the design and implementation of these services.

A systematic tendency of including and excluding certain cost items in the analysis can be a source of potential limitation in economic studies where the findings are skewed towards a particular perspective, for example, the health systems perspective rather than the societal perspective. This will also impact on a balanced assessment of cost-effectiveness of telehealth in rural and remote emergency departments. Due to the small number of economic evaluations and the varied research methodology, the scope for quantitative meta-analysis may be restricted. By confining to articles written in the English language, this analysis may be subjected to publication bias and results need to be interpreted accordingly.

In the event of any difference between the protocol and the complete review, these amendments will be documented including the date of amendment, description of change, rationale, and consequences of these modifications.

## Supplementary information

**Additional file 1.** Quality Assessment Tool PRISMA-P 2015 Checklist.

**Additional file 2.** Search Strategy/Search Concept Grid.

**Additional file 3.** Data Extraction Items and Descriptions.

**Additional file 4.** Column Headings for Study Level Summary Tables.

**Additional file 5.** Article Categorisation Count/Effectiveness Measure Collection Matrix.

## Data Availability

Data sharing is not applicable to this article as no dataset were generated or analysed during the current study.

## Abbreviations

PROSPERO: International Prospective Register of Systematic Reviews
PICO: Participants, Intervention, Comparators and Outcomes
PCC: Population, Concept, Context
AMED: Allied Health and Complementary Medicine
CINAHL: Cumulative Index to Nursing and Allied Health Literature
MeSH: Medical Subject Heading
PRISMA: Preferred Reporting Items for Systematic Reviews and Meta-Analysis
CHEERS: Consolidated Health Economics Evaluation Reporting Standards
ICER: Incremental cost-effectiveness ratio
