# Supplementary material for: Effectiveness and cost-effectiveness of telehealth in rural and remote emergency departments: a systematic review protocol
Source: Syst Rev. 2020 Apr 17;9:82. doi: 10.1186/s13643-020-01349-y (PMC7164257; doi:10.1186/s13643-020-01349-y)
Supplement: Supplementary file 2 — Additional file 2. Search Strategy/Search Concept Grid. [file 13643_2020_1349_MOESM2_ESM.docx]

**Additional File 2 Search Strategy / Search Concept Grid**

| **Concept 1 telehealth** | **Concept 2 effectiveness** | **Concept 3 cost-effectiveness** | **Concept 5 rural or remote** |  |
| --- | --- | --- | --- | --- |
| tele* AND health care or "Delivery of Health Care"/ | effectiveness.mp. | cost-effectiveness.mp. | Rural Health/ | OR |
| Remote Consultation/ | Comparative Effectiveness Research/ | Cost-Benefit Analysis/ | rural.mp. |  |
| Telemedicine/ | clinical effectiveness.mp. | economic evaluation.mp. | Hospitals, Rural/ |  |
| telehealth | Treatment Outcome/ | cost-utility analysis.mp. | Rural Population/ |  |
| eHealth, or mHealth |  | cost effectiveness.mp. | remote.mp. |  |
| “mobile health” or “digital health” |  |  | Rural Health Services/ |  |
|  | OR | |  |  |
|  | AND | |  |  |
