# Supplementary material for: Effectiveness and cost-effectiveness of telehealth in rural and remote emergency departments: a systematic review protocol
Source: Syst Rev. 2020 Apr 17;9:82. doi: 10.1186/s13643-020-01349-y (PMC7164257; doi:10.1186/s13643-020-01349-y)
Supplement: Supplementary file 3 — Additional file 3. Data Extraction Items and Descriptions. [file 13643_2020_1349_MOESM3_ESM.docx]

**Additional File 3 Data Extraction Items and Descriptions**

| Data Collection Item | Item description / sub-groups |
| --- | --- |
| Bibiliographic Information | |
| Author |  |
| Title |  |
| Year of publication |  |
| Country |  |
|  | |
| Methods | |
| Intervention |  |
| Aim |  |
| Objectives |  |
| Participant characteristics | Intervention group |
|  | Comparator details |
| Outcome measures | Clinical effectiveness |
|  | Health service utilisation indicators |
|  | Other effectiveness measures |
|  | |
| Additional Data Item for Economic Evaluations | |
| Perspectives of economic analysis |  |
| Cost items |  |
| *Direct medical costs* | *For each cost item note:*   - *adjustments made* - *data collection issues* - *items omitted and why* |
| *Direct non-medical costs* |  |
| Indirect costs |  |
| Economic models used |  |
| ASSUMPTIONS |  |
| Sensitivity analysis |  |
|  | |
| Results | |
| Study parameters | The values, ranges, references, and, if used, probability distributions for all parameters. Reasons or sources for distributions used to represent uncertainty where appropriate. |
| Incremental costs and outcomes | Mean values for the main categories of estimated costs and outcomes of interest, as well as mean differences between the comparator groups. Incremental cost-effectiveness ratios. |
| Characterising uncertainty | *Single study-based economic evaluation: t*he effects of sampling uncertainty for the estimated incremental cost and incremental effectiveness parameters, together with the impact of methodological assumptions (such as discount rate, study perspective). |
|  | *Model-based economic evaluation:* the effects on the results of uncertainty for all input parameters, and uncertainty related to the structure of the model and assumptions. |
| Characterising heterogeneity | The differences in costs, outcomes, or cost-effectiveness that can be explained by variations between subgroups of patients with different baseline characteristics or other observed variability in effects that are not reducible by more information. |
|  | |
| Discussion | |
| Study findings, limitations, generalisability, and current knowledge | Summarise key study findings and describe how they support the conclusions reached. Discuss limitations and the generalisability of the findings and how the findings fit with current knowledge. |
