# Supplementary material for: Effectiveness and cost-effectiveness of telehealth in rural and remote emergency departments: a systematic review protocol
Source: Syst Rev. 2020 Apr 17;9:82. doi: 10.1186/s13643-020-01349-y (PMC7164257; doi:10.1186/s13643-020-01349-y)
Supplement: Supplementary file 4 — Additional file 4. Column Headings for Study Level Summary Tables. [file 13643_2020_1349_MOESM4_ESM.docx]

Additional File 4 Column Headings for Study Level Summary Tables

| Summary Tables | Column Headings |
| --- | --- |
| Bibliographic Information | Specialty; Author, Year, Title; Country, Study type, Aim, Objective |
| Intervention | Specialty, Author, Year, Title, country, Intervention, Characteristics of the Region (including distance from regional or tertiary centres) |
| Outcomes | Specialty, Author/Year, Object of Comparison, Study Type, Primary Outcome, Data Collection, Secondary Outcome, Data Collection |
| Conclusions | Specialty, Author/Year, Object of Comparison, Intervention Group, Comparator Group, Conclusion, Additional Research |
