# Supplementary material for: Effectiveness and cost-effectiveness of telehealth in rural and remote emergency departments: a systematic review protocol
Source: Syst Rev. 2020 Apr 17;9:82. doi: 10.1186/s13643-020-01349-y (PMC7164257; doi:10.1186/s13643-020-01349-y)
Supplement: Supplementary file 5 — Additional file 5. Article Categorisation Count/Effectiveness Measure Collection Matrix. [file 13643_2020_1349_MOESM5_ESM.docx]

**Additional File 5 Article Categorisation Count / Effectiveness Measure Collection Matrix**

|  | Diagnostic | Direct Consultation | Specialist Support | Monitoring |
| --- | --- | --- | --- | --- |
| General (non-specialty specific) |  |  |  |  |
| Stroke |  |  |  |  |
| Cardiovascular |  |  |  |  |
| Trauma |  |  |  |  |
| Paediatrics |  |  |  |  |
| Mental Health |  |  |  |  |
| Ophthalmology |  |  |  |  |
| Respiratory medicine |  |  |  |  |
| Minor injury |  |  |  |  |
| Burns |  |  |  |  |
| Musculoskeletal |  |  |  |  |
